# Mutagenicity of *Tectona grandis* Wood Extracts and Their Ability to Improve Carbohydrate Yield for Kraft Cooking Eucalyptus Wood

**DOI:** 10.3390/molecules26237171

**Published:** 2021-11-26

**Authors:** Yulia Anita, Syelvia Putri Utami, Hiroshi Ohi, Evelyn Evelyn, Akiko Nakagawa-Izumi

**Affiliations:** 1 Faculty of Life and Environmental Sciences, University of Tsukuba, 1-1-1 Tennodai, Tsukuba 305-8572, Ibaraki, Japan; yuliaanita.lipi@gmail.com (Y.A.); syelvia.putriutami@lecturer.unri.ac.id (S.P.U.); oi.hiroshi.gm@u.tsukuba.ac.jp (H.O.); 2 Research Center for Chemistry, National Research and Innovation Agency Republic of Indonesia, Puspiptek Area, Serpong 15314, Indonesia; 3 Faculty of Engineering, Campus Bina Widya, School of Pulp and Paper Technology, University of Riau, Pekanbaru 28293, Indonesia; evelyn@eng.unri.ac.id

**Keywords:** teak wood, mutagenicity, wood extractives, 2-methylanthraquinone, deoxylapachol, kraft pulp, lignin, carbohydrate

## Abstract

Considering the toxicity of the impurities of synthesized anthraquinone, this study clarified new catalytic compounds for kraft cooking with improved carbohydrate yield and delignification and less mutagenicity, which are important for ensuring the safety of paper products in contact with food. The 2-methylanthraquinone contents of teak (*Tectona grandis*) woods were 0.18–0.21%. Acetone extracts containing 2-methylanthraquinone from Myanmar and Indonesia teak woods as additives improved lignin removal during kraft cooking of eucalyptus wood, which resulted in kappa numbers that were 2.2–6.0 points lower than the absence of additive. Myanmar extracts and 2-methylanthraquinone improved carbohydrate yield in pulps with 1.7–2.2% yield gains. Indonesia extracts contained more deoxylapachol and its isomer than 2-methylanthraquinone. The residual content of 2-methylanthraquinone in the kraft pulp was trace. Although Ames tests showed that the Indonesia and Myanmar extracts were mutagenic to *Salmonella typhimurium*, 2-methylanthraquinone was not. The kraft pulp obtained with the additives should be safe for food-packaging applications, and the addition of 0.03% 2-methylanthraquinone to kraft cooking saves forest resources and fossil energy in industries requiring increased pulp yield.

## 1. Introduction

One of the first applications of anthraquinone (AQ) (9,10-anthracenedione) or AQ derivatives as an additive in alkaline cooking was reported in 1972 by Bach and Fiehn [1]. AQ is known to increase the rate of delignification; reduce cooking time, temperature, or chemical charge; increase pulp yield. Thus, AQ has beneficial effects in the pulp and paper industries for incremental pulp production, energy reduction, and protection of wood resources [2]. The catalytic effect of AQ is described as a redox cycle in which AQ is reduced to anthrahydroquinone (AHQ) by carbohydrates in the wood. The AHQ is then oxidized back to AQ via a reaction with lignin, reducing the active site (ß-*O*-4) in lignin during alkaline cooking [3].

Most kraft pulp mills in Japan use soluble anthraquinone (SAQ) at doses of 0.02–0.1% (200–1000 mg/kg) based on oven-dried wood weight [4]. The US Food and Drug Administration (FDA) allows an AQ charge at the level of 0.1% on oven-dried wood as an additive during the alkaline cooking of wood material, which is the maximum amount for paper that will contact food [2].

Derivatives of AQ have been identified in the leaves, flowers, wood, bark, roots, and fruits of various higher plants as well as in fungi, marine animals, and insects [5,6]. The mycelium of *Pyrenophora graminea* consists of two oxygenated AQ molecules, and these structures do not exert catalytic effects on cooking [6].

Anthraquinone can also be synthesized via the oxidation of anthracene, the Friedel–Craft reaction, and Diels–Alder chemistry [7]. Some AQ derivatives, such as 2-methylanthraquinone (2-MAQ), are also effective cooking additives [8].

Tectoquinone (natural 2-MAQ) has been found in teak (*Tectona grandis* L. f.) wood. Teak grows naturally in Southeast Asia, and it has been introduced into other tropical and subtropical regions because of its excellent tolerance to seasonal drought [9]. Teak wood is one of the most important tropical hardwoods and a highly valued hardwood in Indonesia [10]. Teak has traditionally been grown during rotations of 80–100 years in Java, Indonesia, but this has now been shortened to 18–25 year-rotations [11].

Naphthoquinones and AQs are major secondary metabolites in teak wood [12]. Teak wood extracts mainly contain lapachol, deoxylapachol, 5-hydroxylapachol, 2-MAQ, 1-hydroxy-2-MAQ, obtusifolin, betulinic acid, trichione, and squalene [13]. One report describes that AQ and its derivatives, such as 2-MAQ, can serve as additives for the pulp industry [6].

Generally, 2-MAQ is synthesized from 1,4-naphthaquinone and isoprene via the Diels–Alder reaction, and it can also be synthesized from phthalic anhydride and toluene by the Friedel–Crafts reaction. Here, we used synthesized 2-MAQ with Myanmar and Indonesia teak wood extracts containing natural 2-MAQ as additives in the kraft cooking of *Eucalyptus globulus* wood.

The purity of the final products of industrial AQ generated via anthracene oxidation, the Friedel–Craft reaction, and Diels–Alder chemistry varies [7]. How AQ products are synthesized is important to understand when assessing their biological activity, because potential contaminants are inherent within synthetic processes. 

In addition, traces of hazardous chemicals that remain in cellulosic materials can be transferred from paper packaging or cardboard manufactured for foodstuffs when food is packed with paper [14]. Some impurities in AQ, such as 9-nitroanthracene (9-NA) and 1-hydroxy- and 2-hydroxyAQs (HAQs), can exert serious negative effects in humans and have the potential of being carcinogenic and mutagenic [7]. The International Agency for Research on Cancer (IARC) has classified AQ along with the HAQs as possibly carcinogenic to humans “Groups 2B” and 9-NA as “Groups 3”. On the other hand, the German Federal Institute for Risk Assessment (BfR) delisted AQ as chemicals approved for manufacturing paper and paperboard in contact with food in February 2013 [3,14]. The National Toxicology Program (NTP) report on the oxidation of AQ via the anthracene route described AQ as inducing a weak to modest increase in tumors in the kidneys and bladders of male and female F344/N rats and a modest increase in the livers of male and female B6C3F1 mice [15]. Thus, the use of AQ is regulated in the paper industry, and it has since decreased [14].

Considering the toxicity of the impurities of synthesized AQ, the present study aimed to determine new catalytic compounds for kraft cooking to improve carbohydrate yield and delignification and to evaluate residual amounts of these compounds in cellulosic materials and their mutagenicity to ensure the safety of paper products in contact with food.

## 2. Results and Discussion

### 2.1. Chemical Composition of Eucalyptus Globulus Wood

The results of the chemical analysis indicated that *E. globulus* consisted of 47.6% glucan, 15.3% xylan, 27.7% total lignin with acid insoluble and soluble lignin of 6% and 21.7%, respectively, and 0.93% acetone extractives (Table 1).

Compared with the reported contents of lignin and acetone extracts of *Acacia crassicarpa* wood (31.5% and 2.08%, respectively) [16], these were low, and nitrobenzene oxidation of *E. globulus* wood resulted in a syringaldehyde (S)/vanillin (V) ratio of 4.60 and 2.22 mmol/g of lignin for S and V yields, respectively, indicating that the lignin had a more syringyl and non-condensed structure. The ratio of xylan to glucan in *E. globulus* (0.321) was higher than that of *A. crassicarpa* (0.265), which is advantageous for producing kraft pulp for paper materials.

### 2.2. Contents of Natural 2-Methylanthraquinone in Myanmar and Indonesia Teak Wood

**Figure 1:**
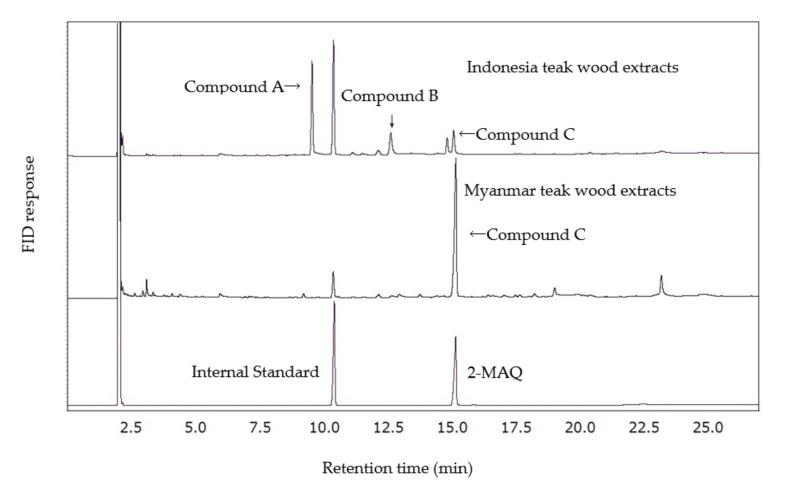
Gas chromatogram of Indonesia and Myanmar teak wood extracts.

Analysis of Indonesia teak wood extracts by gas chromatography (GC) revealed the main compounds **A**, **B**, and **C** at retention times of 9.6, 12.7, and 15.1 min, respectively (Figure 1). 

Compound **C** was identified as 2-MAQ by comparison with authentic 2-MAQ using GC/MS. Thus, the 2-MAQ content determined from the calibration curve was 0.18% based on the wood weight for Indonesia teak wood (Table 2) compared with 0.21% in Myanmar teak wood [17].

We identified two major unknown compounds in Indonesia teak wood extracts using GC/mass spectrometry (MS). The mass spectra of compounds **A** and **B** were isomers based on their apparent molecular ion peaks at 226. Indonesia teak wood contained deoxylapachol and isodeoxylapachol, and it had a molecular mass of 226 [13,18,19]. The contents of compounds **A** and **B** were 20% of the Indonesia teak wood acetone extracts based on the peak areas of FID (GC-2014S) response, which should be 7.8-fold the 2-MAQ content. As shown in Figure 1, the contents of compounds **A** and **B** in the Myanmar extracts were trace and probably less than one-80th (1.25%) of those of the Indonesia extracts.

### 2.3. Degree of Residual Lignin and Carbohydrate Yield in Kraft Pulps

#### 2.3.1. Effects of Teak Wood Extracts as Additives on Kraft Cooking

When used as cooking additives, kappa numbers with additives were lower than those without Myanmar and Indonesia teak wood extracts and chemically synthesized 2-MAQ (Table 3). 

The kappa numbers after kraft cooking with teak wood extracts (14.8–18.7) decreased at a 16–17% active alkali dose compared to those with no additive at the same active alkali (19.9 and 22.6). The decrease was in the order of Myanmar teak wood extracts > Indonesia teak wood extracts ≥ 2-MAQ. The acetone extracts of teak wood contained not only natural 2-MAQ but also other aromatic compounds. If the beneficial cooking catalyst effect of the Myanmar extracts was caused by natural 2-MAQ alone, then we would not be able to explain the better effect over 2-MAQ and, therefore, we expect that the beneficial effect comes from both natural 2-MAQ and other aromatic compounds in the extracts.

Kraft cooking with Myanmar teak wood extracts increased pulp yield by 1.7–2.2%. Adding an additive, such as 0.03% 2-MAQ, to kraft cooking saves forest resources and fossil energy in industries, as it increases pulp yields compared with the absence of the additive [20]. 

#### 2.3.2. Effects of Additives on Carbohydrate Yield for Kraft Cooking

**Figure 2:**
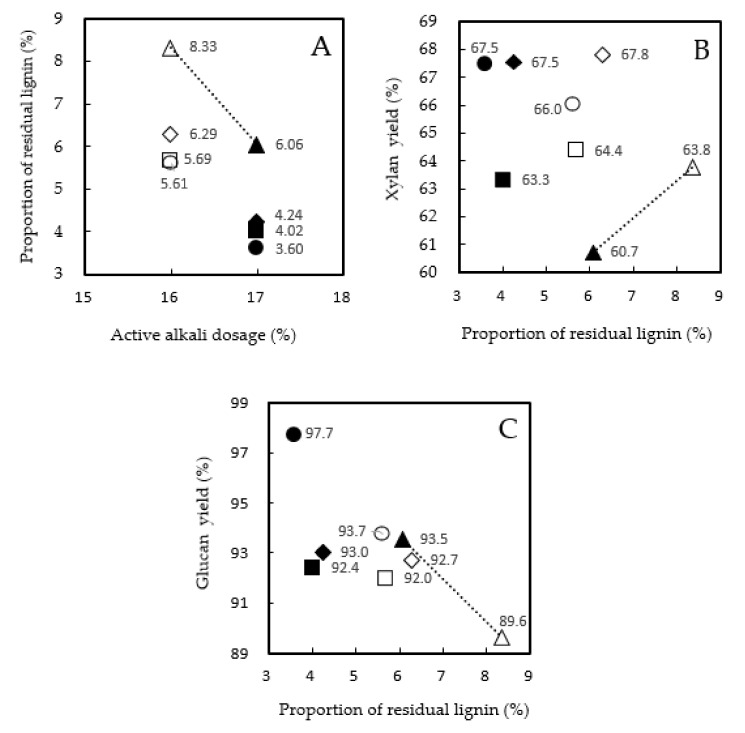
(**A**) Proportion of the residual lignin; (**B**) xylan yield; (**C**) glucan yield in pulps obtained by kraft cooking. ●: Myanmar teakwood extracts; □: Indonesia teakwood extracts; ◇: 2-methylanthraquinone; △: without additives. The white color represents a 16% active alkali dose; the black color represents a 17% active alkali dose.

The contents of acid-insoluble lignin, acid-soluble lignin, glucan, and xylan in pulp samples were analyzed. The content of residual lignin (%) was the sum of acid-insoluble and acid-soluble lignin (%) in pulp and was converted by pulp yield into residual lignin (%) based on the wood weight. The proportion of residual lignin (%) in Figure 2 was obtained by the residual lignin (%) ÷ lignin content of wood (27.7%) × 100, and Figure 2A shows that the effects of facilitating lignin degradation were more evident in the order of Myanmar teak wood extracts > Indonesia teak wood extracts ≥ 2-MAQ. 

Glucose and xylose in the pulp hydrolysate were converted into polymeric glucan and xylan (%) based on the pulp weight, then further converted by pulp yield into the yields (%) in pulp based on wood weight. Figure 2B,C show the xylan and glucan yields obtained from the xylan (%) of the pulp ÷ the xylan content of the wood (15.3%) × 100 and from the glucan (%) of the pulp ÷the glucan content of the wood (47.6%) × 100. Figure 2B clearly shows the effects of the additives on the xylan yields of the kraft pulp. Myanmar and Indonesia teak wood extracts contain 2-MAQ; thus, the results here showed no differences for all additive compounds. Figure 2C shows similar glucan yields with and without additives.

The catalytic effect of AQ is described as a redox cycle in which AQ is reduced to AHQ by the carbohydrate in wood. The AHQ is then oxidized back to AQ in a reaction with lignin, then reduced via the ß-*O*-4 bond in the lignin during alkaline cooking [3]. Xylan is unstable under extreme alkaline conditions during kraft cooking, whereas additives, such as 2-MAQ, can oxidize the reduced end group of the xylan polymer in a similar way as AQ and, consequently, depress the peeling reaction (degradation) of the xylan polymer. Therefore, the xylan yield improved. Glucan, which consists of highly crystalline cellulose, is more stable than xylan under kraft cooking conditions; therefore, a clear effect was not evident.

### 2.4. Residual Content of 2-Methylanthraquinone in Kraft Pulps

The residual content of 2-MAQ in kraft pulp was 1.70–6.00 mg/kg (Table 4).

The residual 2-MAQ decreased as the kappa number decreased, because 2-MAQ was more easily adsorbed by hydrophobic dissolved lignin in the spent (black) liquor than by the hydrophilic carbohydrate-rich pulp. Most 2-MAQ was found in black liquor at the end of the cooking period and was almost completely adsorbed or chemically bound to lignin in solution. The residual 2-MAQ should be maintained at low levels in unbleached pulp after sufficient washing during pulp and paper production at mills, when 2-MAQ is added to kraft cooking. 

The quantity of 2-MAQ transferred to food is 0.01 mg/kg of food when the maximum amount of residual 2-MAQ in pulp is 8 mg/kg [17]. The above results showed a maximum residue of 6.00 mg/kg, indicating that the quantity of 2-MAQ transferred to food was a maximum of only 0.0075 mg/kg.

Determining the residual 2-MAQ in bleached pulp used for food wrapping and packaging materials is also important. A previous study [17] clarified that the residual 2-MAQ content in oxygen-bleached pulp was 0.2–0.3 mg/kg based on pulp weight. No residual 2-MAQ was detected in the final bleached pulp determined by GC/MS [17]. 

These results showed that trace amounts of 2-MAQ remaining in the pulp in combination with residual lignin can be reduced to non-detectable levels by pulp bleaching.

### 2.5. Mutagenicity of Teak Wood Extracts: S. typhimurium Ames Tests

Table 5 shows the results of bacterial reverse mutation tests using *S. typhimurium* strains TA100 and TA98 with 10% rat S9. 

Indonesia teak wood extracts increased the number of revertant colonies to at least twice as much as the negative control for the strains with metabolic activation, and dose-dependence and reproducibility were confirmed. The test substance was mutagenic under the conditions of the Ames test.

The acetone extracts of Myanmar teak wood were mutagenic (positive) against *S. typhimurium strain* TA 100 with 10% rat S9, but the response to strain TA 98 was not detectably mutagenic [17]. Figure 1 shows that the contents of deoxylapachol and its isomers (compounds **A** and **B**) in Myanmar extracts were trace and probably less than one-80th (1.25%) of those of Indonesia extracts. Therefore, the mutagenic effects of Myanmar and Indonesia extracts should result in the generation of other compounds in the extracts, but not deoxylapachol and its isomer. 2-MAQ prepared by the Diels-Alder reaction was negative towards TA 100 and TA 98 with 10% rat S9 at up to 10,000 μg/plate [17]. These findings suggested that native 2-MAQ in the teak extract was not mutagenic. According to Wilson et al., 2-MAQ is a potent inducer of aryl hydrocarbon receptor (AhR) signaling which may mediate toxic effect [21]. To date, there are still contradicted results on the toxicity of 2-MAQ which probably due to the impurities of the 2-MAQ used from different production methods when assessing their biological activity in different investigations. Based on our Ames test results, 2-MAQ obtained by Diels Alder reaction was not mutagenic, therefore its addition in kraft cooking would be unlikely to cause genotoxic effect.

## 3. Materials and Methods

### 3.1. Materials

Myanmar and Indonesia teak woods were used as cooking additives. Disks of Myanmar and Indonesia teak woods were obtained and collected from HOXAN Corporation, Tokyo, Japan, and the local timber company: Mabel jati surya indah, Jepara, Indonesia, respectively. Each species of teak wood was cut into small pieces and then ground into 40–80 mesh wood meal. Acetone (FUJIFILM Wako Pure Chemical Corporation, Tokyo, Japan) was used for wood meal extraction. The 2-MAQ (98% purity) used as a cooking additive was prepared by a Diels–Alder reaction and provided by Kawasaki Kasei Chemicals, Kawasaki City, Japan. Teak wood acetone extracts and 2-MAQ were used independently as cooking additives. We also analyzed the mutagenicity of Indonesia teak wood extracts.

*Eucalyptus globulus* wood chips from a Chilean plantation were provided by a pulp mill (Hokuetsu Corporation, Tokyo, Japan) and used as material for kraft cooking with the addition of teak extracts or 2-MAQ.

### 3.2. Soluble 2-MAQ Preparation

Soluble 2-MAQ, as a cooking additive, was prepared by dissolving 100 mg of 2-MAQ in 10 mL of 2 M NaOH containing 200 mg of glucose in sealed vials at 95–100 °C for 1 h. Samples of teak wood extracts were similarly processed before kraft cooking.

### 3.3. Kraft Cooking

*Eucalyptus globulus* wood chips (50 g, oven dried) were placed in a stainless-steel reactor and kraft cooked with 2-MAQ in 250 mL of liquor (5 mL/g of wood) for 3 h at 145 °C. The amount of added 2-MAQ was 0.03% based on the weight of the oven-dried wood chips. Individually, the dose of Myanmar or Indonesia teak wood extracts was 1.16% and 1.22%, respectively, based on the wood weight, which contained 2-MAQ with a level of 0.03% on wood. The active alkali doses were 16% and 17%, and the sulfidity was 30%.

### 3.4. Chemical Analysis of Wood and Kraft Pulp

Chemical characterizations of *E. globulus* wood meal, such as acid-insoluble lignin (Klason lignin), ash content, and extractives were analyzed based on TAPPI test methods (T222 om-11, T211 om-93, and T204 om-88, respectively). For monosaccharide (i.e., glucose and xylose) analysis, wood meal was hydrolyzed with 72% sulfuric acid followed by 4% sulfuric acid for 1 h at 121 °C [22]; then, the resulting filtrate was analyzed using an ICS 3000 ion chromatograph (Dionex GmbH, Lohmar, Germany). The acid-soluble lignin content in the filtrate was determined according to TAPPI test method T222 om-88 using UV–Vis spectrophotometry at 205 nm. Yields of syringyl aldehyde (S) and vanillin (V) and the S/V molar ratio were determined in wood meal oxidized using nitrobenzene as described [22].

The pulp characterization was determined with the kappa number as a measure of the degree of the delignification in the cooking process by following TAPPI test method T236 om-13.

### 3.5. Extraction of Teak Wood and Determination of Residual 2-MAQ in Pulp

Oven-dried wood meal (5 g) and unbleached kraft pulp (2.3 g o.d.) were mixed with 150 mL of acetone for 8 h in a Soxhlet extractor; then, the extracts were rotary evaporated to near dryness.

The amounts of 2-MAQ in teak wood extracts and unbleached pulp were measured by GC using a GC-2014S system (Shimadzu Corporation, Kyoto, Japan) equipped with a DB-1 column (30 m × 0.25 mm i.d.; film thickness, 0.25 μm) and a flame ionization detector (FID) with helium as the carrier gas. The injector and detector temperatures were 300 and 280 °C, respectively. Samples of teak wood extracts were injected in a split ratio of 50:1, and unbleached pulp was injected in the split-less mode. The temperature profile was as follows: 160 °C for 5 min, 160–200 °C in 10 °C/min increments for 4 min, 200–280 °C in 10 °C/min increments for 8 min, and 280 °C for 4 min. The 2-MAQ content of each sample was calculated from a calibration curve of *n*-eicosane as the internal standard.

We identified 2-MAQ and compounds **A** and **B** by GC-MS using a GC-MS apparatus QP-5050 system (Shimadzu Corporation, Kyoto, Japan) equipped with a DB-5 MS column (30 m × 0.25 mm i.d.; film thickness, 0.5 μm) and operated in the electron impact mode (70 eV) with helium as the carrier gas. The injection and ion source temperatures were 280 and 210 °C, respectively, and the split ratio was 21. The GC temperature profile was 220 °C for 12 min, 220–240 °C in 5 °C/min increments for 4 min, 2 min at 240 °C, 240–280 °C in 10 °C/min increments for 4 min, and 280 °C for 4 min. The products were determined by comparing their retention times and mass spectral data with those of authentic compounds.

### 3.6. Mutagenicity of Teak Wood Extracts: Salmonella typhimurium Ames Tests

This study was designed to assess the mutagenic potential of wood extractives of *Tectona grandis* using a bacterial reverse mutation test system with *Salmonella typhimurium* strains TA 100 (for base-pair exchange mutation) and TA 98 (for frameshift mutation) [23]. The mutagenicity of Indonesia teak wood extracts with a 2-MAQ content of 2.58% was assayed using Ames tests of *S. typhimurium*. strains T98 and T100 in the presence of rat S9 fraction (UBE Scientific Analysis Laboratory, Inc., Ube, Japan). A positive response was indicated by at least a two-fold increase in mean revertants per plate versus that of the vehicle control [23,24]. This test was conducted according to the OECD Principles of Good Laboratory Practice (as revised in 1997) and the OECD Guideline for Testing of Chemicals (21 July 1997), 471: Bacterial Reverse Mutation Test. Myanmar extract for mutagenicity was previously reported [17].

## 4. Conclusions

Extracts of Myanmar and Indonesia teak woods containing 2-MAQ improved kappa number decreases by removing lignin during kraft cooking compared with the absence of an additive. Extracts with 2-MAQ increased pulp yields by 1.7–2.2% and increased xylan yields in pulp. Extracts of Indonesia teak wood contained more deoxylapachol and its isomer than 2-MAQ. Only trace amounts of 2-MAQ residues were detected in kraft pulp. Whereas Indonesia and Myanmar extracts were mutagenic towards *Salmonella* *typhimurium* in Ames tests, 2-MAQ was not. The kraft pulp obtained with the additives should be safe for food-packaging applications, and the inclusion of 0.03% 2-MAQ during kraft cooking saves forest resources and fossil energy in industries concerned with increasing pulp yield. Considering the toxicity of impurities arising during anthraquinone synthesis, we proposed a novel catalytic compound for kraft cooking that is neither genotoxic nor mutagenic, and it can improve carbohydrate yield and delignification, which are important for ensuring the safety of paper products that will come into contact with food.

## Figures and Tables

**Table 1:** Chemical composition of *Eucalyptus globulus* wood.

Components (%)	
Glucan	47.6 ± 1.8
Xylan	15.3 ± 0.5
Other sugars	1.08 ± 0.02
Total lignin	27.7 ± 0.2
Ash	0.47 ± 0.05
Acetone extracts	0.93 ± 0.03
Unknown	6.92
Total (%)	100
Nitrobenzene oxidation	
Syringaldehyde (S)/vanillin (V) molar ratio	4.60 ± 0.21
S and V yields (mmol/g lignin)	2.22 ± 0.05

**Table 2:** Contents of natural 2-methylanthraquinone in Myanmar and Indonesia teak woods.

	Acetone Extracts	2-Methylanthraquinone	
	(%)	(%, in Acetone Extracts)	(%, in Wood)
Myanmar teak wood	7.95 ± 0.06	2.59 ± 0.20	0.21 ± 0.02
Indonesia teak wood	6.95 ± 0.39	2.58 ± 0.10	0.18 ± 0.01

**Table 3:** Effects of teak wood extracts and 2-methylanthraquinone on kraft cooking.

	Active Alkali Dose (%)	Kappa Number	Pulp Yield (%)
Myanmar teak wood extracts 1.16% dosage	16	16.6 ± 0.1	58.8 ± 0.5
	17	14.8 ± 0.7	58.3 ± 0.4
Indonesia teak wood extracts 1.22% dosage	16	18.7 ± 0.6	55.6 ± 0.3
	17	17.7 ± 0.6	55.4 ± 0.3
2-Methylanthraquinone 0.03% dosage	16	19.6 ± 1.2	57.3 ± 0.3
	17	17.1 ± 1.3	56.8 ± 0.3
No additive	16	22.6 ± 0.1	57.1 ± 0.1
	17	19.9 ± 0.3	56.1 ± 0.1

**Table 4:** Residual 2-methylanthraquinone (2-MAQ) contents in kraft pulp.

	Active Alkali Dose (%)	Kappa Number	Residual 2-MAQ (mg/kg)
Myanmar teak wood extracts	16	16.6	2.42 ± 0.62
	17	14.8	1.70 ± 1.54
Indonesia teak wood extracts	16	18.7	2.86 ± 0.09
	17	17.7	3.61 ± 0.07
2-Methylanthraquinone	16	19.6	6.00 ± 1.42
	17	17.1	4.79 ± 1.46

**Table 5:** Mutagenicity of Indonesia teak wood extracts and 2-methylanthraquinone (2-MAQ) in *Salmonella typhimurium* Ames tests.

Strain	Dose of Extracts or 2-MAQ (μg/Plate)	Revertants/Plate + 10% Rat S9			
		Indonesia Extracts		Myanmar Extracts [20]	2-MAQ [20]
		1st Test	2nd Test		
TA 100	0	109 ± 10	112 ± 9	98 ± 13.7	98 ± 13.7
	156	145 ± 20	151 ± 14	117 ± 9.0	- ^d^
	313	159 ± 22	165 ± 6	119 ± 10.1	108 ± 6.0 ^c^
	625	169 ± 22	208 ± 6	126 ± 5.9	- ^d^
	1250	221 ± 29	226 ± 19	117 ± 6.9	101 ± 9.1 ^c^
	2500	139 ± 22	144 ± 9	150 ± 8.7	103 ± 9.2 ^c^
	5000 ^a^	79 ± 37 ^b^	0 ± 0 ^b^	199 ± 2.1	114 ± 3.2 ^c^
Trial summary		Positive	Positive	Positive	Negative
Positive control ^e^	1.0	1611 ± 33	1464 ± 80	1566 ± 76.9	1566 ± 76.9
TA98	0	21 ± 2	31 ± 5	23 ± 1.0	23 ± 1.0
	156	25 ± 2	27 ± 5	24 ± 3.5	- ^d^
	313	30 ± 8	32 ± 4	27 ± 6.0	30 ± 7.6 ^c^
	625	39 ± 13	45 ± 5	30 ± 6.1	- ^d^
	1250	55 ± 17	65 ± 3	34 ± 4.0	26 ± 2.9 ^c^
	2500	74 ± 7	75 ± 12	31 ± 6.1	27 ± 6.6 ^c^
	5000 ^a^	0 ± 0 ^b^	0 ± 0 ^b^	28 ± 9.2	33 ± 3.2 ^c^
Trial summary		Positive	Positive	Negative	Negative
Positive control ^e^	0.5	746 ± 71	785 ± 33	570 ± 20.2	570 ± 20.2

^a^ Precipitation of the test material was observed. ^b^ Growth inhibition of bacteria was observed. ^c^ Doses were 300, 1000, 3000, and 10,000 (μg/plate), and precipitation of the test material was observed. ^d^ Doses were not performed at 156, and 625 (μg/plate). ^e^ 2-Aminoanthracene.

## Data Availability

FINAL REPORT—“Bacterial Reverse Mutation Test of Wood Extractives of *Tectona grandis*”, UBE Scientific Analysis Laboratory, Inc., (USA-R-20268) is available.
